# “The Ameliorative Effect of Interleukin-17A Neutralization on Doxorubicin-Induced Cardiotoxicity by Modulating the NF-κB/NLRP3/Caspase-1/IL-1β Signaling Pathway in Rats”

**DOI:** 10.1007/s10753-024-02187-z

**Published:** 2025-03-11

**Authors:** Mostafa D. Hassen, Nahla O. Mousa, Sara M. Radwan, Refaat M. Gabre

**Affiliations:** 1 Department of Biochemistry, Faculty of Pharmacy, Ain Shams University, Cairo, 11566 Egypt; 2 Department of Biotechnology, Faculty of Science, Cairo University, Giza, 12613 Egypt

**Keywords:** Doxorubicin, Interleukin-17A, Secukinumab, Nuclear factor kappa beta, Pyrin domain containing 3, Interleukin-1β

## Abstract

Doxorubicin (DOX) is used as a chemotherapeutic drug for treating cancer. Nevertheless, it causes damage to the heart by activating inflammatory pathways, resulting in cardiotoxicity. Imbalance in cytokine production is a crucial component that might trigger the initiation of inflammatory processes. Inflammatory cytokines could be targeted therapies against cardiovascular diseases (CVDs). Interleukin-17A (IL-17A) is a cytokine that promotes inflammation and stimulates harmful immunological reactions. The objective of the study was to determine the efficacy of secukinumab (SEC), a completely human monoclonal IgG1/κ antibody that targets IL-17A, in ameliorating DOX-induced cardiotoxicity (DIC). We administered 2.5 mg/kg of DOX intraperitoneally to male Wistar rats three times a week for 2 weeks and simultaneously administered 0.9 mg/kg of SEC along with 2.5 mg/kg of DOX injection three times a week for a duration of two weeks. The findings indicated that DOX induced damage to the heart tissue, resulting in a significant rise in indicators of cardiotoxicity (P < 0.001), as well as oxidative stress and inflammation. DIC may have arisen from DOX's activation of the Pyrin domain containing 3 (NLRP3) inflammasome and the nuclear factor kappa beta (NF-κB) pathway. The co-administration of SEC successfully reversed all DOX-induced abnormalities by restoring cardiac functions to their baseline levels, decreasing levels of inflammatory mediators such as IL-17A and interleukin-1β (IL-1β), and improving oxidative stress by reducing levels of malondialdehyde (MDA) and increasing levels of reduced glutathione (GSH). Furthermore, it mitigated the heightened activation of the NF-κB/NLRP3 pathway caused by DOX. This study shows that IL-17A neutralization can prevent DIC by regulating the NF-κB/NLRP3/Caspase-1/IL-1β pathway to be used as potential therapeutic target for CVDs.

## Introduction

Doxorubicin (DOX), an antibiotic derived from anthracycline, has been widely employed in clinical settings as a chemotherapeutic agent to combat various forms of cancer [1]. Upon DOX administration, several intense adverse reactions are encountered including cardiotoxicity which is considered as the main major treatment side effect [2]. Many clinical findings showed that left ventricular dysfunction, dilated cardiomyopathy and heart failure are usually developed upon the completion of DOX treatment protocol [3]. Such complications are triggered by cardiomyocyte apoptosis and myocardial fibrosis occurring due to DOX-associated oxidative stress and mitochondrial damage [4]. To date, the cumulative dose of DOX is the main factor that contributes to the onset of cardiomyopathies induced by cancer therapy [5].

The cardiac remodeling is usually developed via aberrated inflammatory responses post-DOX therapy [6]. The complicated cytokines network constitutes a possible potent mechanistic link between DOX therapy and the deteriorated cardiac function [7]. Intriguingly, Interleukin-17A (IL-17A) is an inflammatory cytokine that exacerbates the cardiovascular system and plays a crucial role in the development of cardiovascular diseases and myocardial infarction [8] and responsible for pathogenesis of multiple autoimmune and inflammatory conditions [9]. In addition, IL-17A upregulation was detected in sepsis, pneumonia, allograft rejection conditions, and notably in cancer [10].

As a defensive reaction from the innate immune system during various diseases and infections or stress conditions, a polymeric structure known as inflammasome composed of sensor proteins, junction molecules, and effectors is assembled [11]. The inflammatory response is triggered by the inflammasome, which releases various inflammatory factors such as Interleukin IL-1β (IL-1β), Interleukin IL-18 (IL-18), and Interleukin-37 (IL-37) [12]. The inflammasome activation is responsible for cardiac hypertrophy, pyroptosis and fibrosis hence they are involved in the pathogenesis of heart failure [13].

The NLRP3 inflammasome stands out as the unique inflammasome due to its nucleotide-binding domain, leucine-rich–containing family, and pyrin domain–containing-3 composition that is expressed in diverse types of cells including neutrophils, lymphocytes, macrophages, microglia, epithelial cells, neurons, osteoblasts and dendritic cells [14].

Activated NLRP3 inflammasomes induce the release of IL-1β and IL-18 which contribute in the pathophysiology of atherosclerosis and coronary heart diseases [15]. IL-17 inhibitors are medications that target IL-17, blocking its inflammatory effects by interfering with the binding of IL-17A to its receptor IL17RA. These inhibitors are commonly used to manage inflammatory conditions in gastroenterology, rheumatology, and dermatology [16]. Monoclonal antibody therapies have shown to be particularly effective among the various inhibition strategies. One such monoclonal antibody, Secukinumab (SEC), is approved for treating psoriasis, psoriatic arthritis, and ankylosing spondylitis, with ongoing research for other autoimmune disorders [17]. Recent studies have highlighted the role of IL-17A in connecting cardiovascular disease (CVDs) with psoriatic inflammation, suggesting that anti-IL-17A therapy could not only benefit skin conditions but also reduce cardiovascular inflammation. Administration of Secukinumab has been shown to improve left ventricular function by reducing levels of oxidative stress markers like malondialdehyde (MDA) and protein carbonyl [18].

While the efficacy of IL-17A antagonists in inflammatory disorders has been well described, those on the cancer- and chemotherapy-associated cardiovascular diseases remain less explored. In this study, the potential protective effect of IL-17A neutralization was assessed in rat model of DOX-induced cardiotoxicity (DIC). SEC anti-inflammatory effect and its potential role in attenuating the cardiotoxic effects of DOX through modulation of NF-κB/NLRP3/Caspase-1/IL-1β axis were elucidated.

## Materials and Methods

### Declaration of Ethics

The work was authorized by Cairo University's ethics committee, and all experimental techniques were approved by the Animal Care and Use (CU-IACUC) Committee. The protocols (Protocol # CU-I /F/15/23) were followed to guarantee that adequate animal care and experimental procedures were completed. The study also complied to the NIH's animal research regulations.

### Drugs and Chemicals

Secukinumab (SEC) was acquired in the form of Cosentyx, a prefilled pen containing 150 mg/mL of the drug, manufactured by Novartis Pharmaceuticals Corporation, United States. Doxorubicin (DOX) was purchased from EMIC United Pharmaceuticals Co., based in Egypt. Biochemicals and substrates were obtained from Biodiagnostic in Egypt.

### Animals

This study comprised male Wistar albino rats that were eight weeks old and weighed between 160 and 180 g. Before the trial, the animals were acclimatized for two weeks in a controlled setting with a humidity level of 55 ± 5% and an ambient temperature of 22 ± 1°C. The rats were fed a standard diet and had constant access to water during the study.

### Methodology

**Fig. 1:**
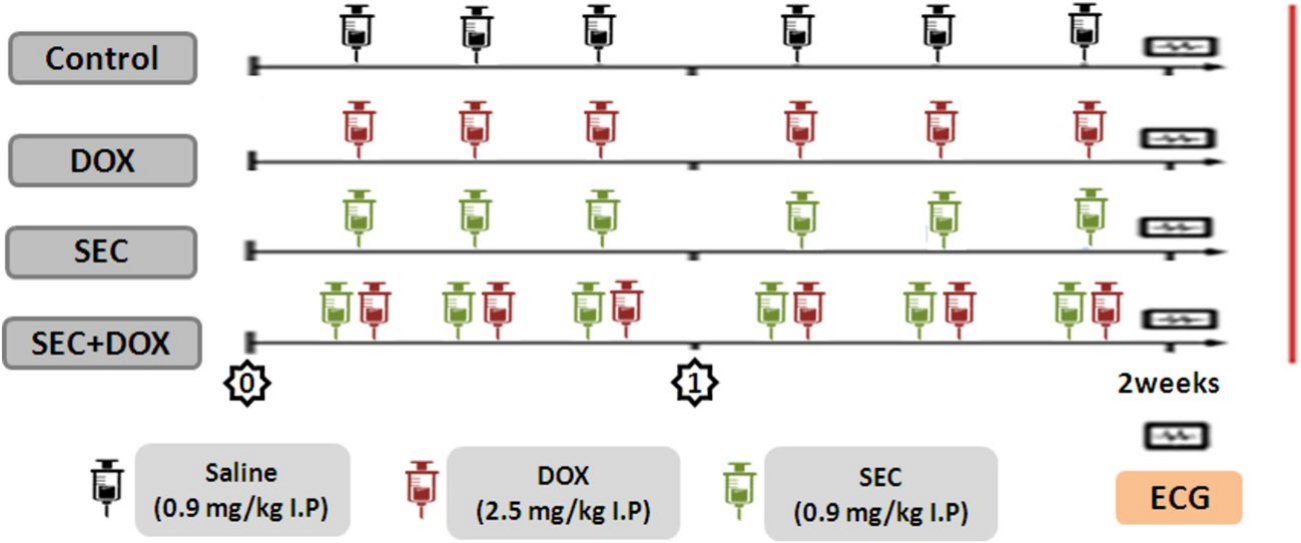
Experimental design and timeline: Rats were allocated into four groups: Control, DOX, SEC, and SEC + DOX groups

A total of twenty-eight Wistar rats were weighed individually before being split into four groups of seven each. The first cohort, known as the control group, was given 0.9 ml/kg of 0.9% saline intraperitoneally three times per week for two weeks. The second group received an intraperitoneal dosage of 2.5 mg/kg DOX three times a week for two weeks, for a total dose of 15 mg/kg [19]. The SEC control group, as stated by [20], was given 0.9 mg/kg SEC intraperitoneally three times each week for two weeks. Finally, the fourth group, SEC + DOX, received 0.9 mg/kg of SEC combined with 2.5 mg/kg of intraperitoneal DOX injection three times each week for two weeks, as seen in (Fig. 1**)** of the study design.

The initial body weight was documented. The animals' overall health and mortality rate were monitored daily for duration of two weeks. The day after the final administration, the animals' final body weights were measured. They were then anesthetized using intraperitoneal injection of phenobarbital (50 mg/kg) and underwent ECG recording. Samples of blood were collected from the retro-orbital plexus and left to coagulate. Subsequently, the samples underwent centrifugation at a speed of 3000 rpm and a temperature of 4°C for duration of 10 min in order to separate the serum. Portions of the serum were then stored at -80°C for subsequent biochemical analysis. The animals were decapitated, and the weights of their hearts were recorded. The hearts were isolated and quickly rinsed with ice-cold saline. A portion of the heart tissues was fixed in 10% phosphate-buffered formalin for 24 h for pathological examination. The remaining portion was swiftly frozen in liquid nitrogen and preserved at -80°C for future examination. Homogenization was carried out using ice-cooled phosphate buffer saline with a pH of 7.4. Subsequently, the mixture was subjected to centrifugation at 10,000 rpm for 10 min at 4°C, and the resultant supernatant was grouped for analysis.

### Electrocardiography (ECG)

An electrocardiogram (ECG) is a diagnostic test that quantifies the electrical activity of the heart. At the start of the inquiry, an ECG was conducted to verify the distinctive ECG pattern of the rats. Following the experiment, the rats were administered phenobarbital at a dosage of 50 mg/kg in order to produce unconsciousness. Subsequently, their ECG was monitored for a duration of 1 min. The heart rate, P duration, QRS Interval, QTc, and ST height were observed using the ECG Power lab module, which includes Power-lab/8sp and Animal Bio-Amplifier from Australia, along with Lab Chart 7 software featuring ECG analyzer [21, 22].

### Body Weight and Ratio of Heart Weight to Body Weight

Body weight was measured at the start and end of the research in each group. The heart weight (HW) to body weight (BW) ratio was also determined [23].

### Histopathological Examination

The heart tissues were conserved by submerging them in a 10% paraformaldehyde solution for 72 h, dehydrated, then embedded in paraffin. Subsequently, the samples were sliced into thin sections measuring 5-μm in thickness. The sections were then treated to remove paraffin and then subjected to staining with haematoxylin and eosin, following recognized techniques for histological assessment. Subsequently, the slides were analyzed using a high-definition light microscope imaging system (Leica Microsystems GmbH, Wetzlar, Germany) to detect and classify various histopathological alterations [24]. Histopathological changes were reported by a score ranging from 0–4 as following: 0 (negative records), 1 (mild records in less than 15% of examined tissue sections), 2 (moderate records in 15–35% of examined tissue sections), 3 (severe records in 36–50% of examined tissue sections) and 4 (very severe records in more than 50% of examined tissue sections).

### Evaluation of Total Protein

The protein level of each homogenized cardiac tissue sample was quantified using the bicinchoninic acid (BCA) protein assay purchased from Abcam in Waltham, MA, USA.

### Evaluation of Oxidative Stress

Oxidative stress levels were evaluated by measuring the amounts of MDA and reduced glutathione (GSH) in cardiomyocyte homogenates. The procedure was conducted in accordance with the instructions supplied by commercially accessible kits (Bio-diagnostic, Cairo, Egypt).

### Evaluation of Serum Biomarkers for Cardiac Toxicity

The activity of blood creatine kinase isoenzyme-MB (CK-MB) was assessed using the spectrophotometric method. To achieve this goal, a commercially available kit obtained from Spectrum Diagnostics, Cairo, Egypt was used. Furthermore, the blood's content of cardiac troponin I (cTn-I) was quantified using an enzyme-linked immunosorbent assay (ELISA) kit (Sun Long Biotech Co, China; Cat. No.: SL0121Mo) in accordance with the manufacturer's instructions.

### Evaluation of Inflammatory Cytokines

The levels of pro-inflammatory cytokines IL-1β and IL-17A were assessed in homogenized samples of cardiac tissue; following the directions provided by the manufacturer of both ELISA kits obtained from the Chinese firm bt-laboratory Co. (Catalogue Number E0119) and the American company AFG Bioscience Co. (Catalogue Number EK720975) respectively.

### Evaluation of Cardiac NLRP3 Level

The NLRP3 level in cardiac tissue homogenate was quantified using ELISA kit (Aviva System Biology Corp, USA; Cat. No.: OKCD04232), following the instructions provided by the manufacturer.

### Evaluation of Nuclear Factor Kappa beta 1 (NF-κB1) Gene Expression

The isolation of total RNA from cardiac tissues was carried out using an Invitrogen R TRIzol reagent Mini kit (Carlsbad, CA), following the manufacturer's instructions. Afterward, the RNA separated was transformed into complementary DNA (cDNA) using the High-Capacity cDNA Reverse Transcription Kit (Applied Biosystems, USA; Cat.No.4368814). The expression level of NF-κB1 was evaluated using the SYBR Green PCR Master Mix (Cat.No. 4309155) from Applied Biosystems, USA in a real-time PCR analysis conducted with an Applied Biosystems Step One Plus thermal cycler. The thermo-cycling parameters recommended by the manufacturer were followed precisely. The technique commenced with an initial denaturation phase, which lasted for a duration of 10 min at a temperature of 95 oC. Afterwards, a sequence of 40 denaturation cycles was carried out at a temperature of 95 oC, with each cycle lasting 30 s. Afterwards, the NFκB1 or GAPDH samples were annealed at temperatures of 55 oC or 56 oC for 30 s. Ultimately, the samples were subjected to a process of elongation at a temperature of 72 oC for a duration of 30 s. The PCR cycle concluded with a final extension step that lasted for 5 min at a temperature of 72 degrees Celsius. The real-time PCR experiment was conducted with an Applied Biosystems Step One Plus thermal cycler. The primer sequences are shown in (Table 1**)**. The data analysis was conducted via the ABI Prism R 7000 SDS software. The relative fold gene expression was calculated by 2^−ΔΔCt^ method. The NFκB1 mRNA levels were standardized using glyceraldehyde 3-phosphate dehydrogenase (GAPDH) as reference genes.

**Table 1:** The nucleotide sequences used as primers for real-time PC

Gene	Primer	Accession number
NFκB1	Forward:5’- AATTGCCCCGGCAT -3' Reverse: 5’- TCCCGTAACCGCGTA -3’	[NM_01276711.1]
GAPDH	Forward: 5’- ATGACTCTACCCACGGCAAG -3' Reverse: 5’- CTGGAAGATGGTGATGGGTT -3’	[NM_017008.3]

### Evaluation of Cardiac Caspase-1 Protein

The immunohistochemistry staining for caspase-1 was conducted in accordance with the manufacturer's procedure. The cardiac sections underwent deparaffinization, rehydration, and antigen unmasking using a 10 mM sodium citrate solution (pH 6.0) at 60°C for 10 min. Afterwards, the slides were chilled for a duration of 30 min. Subsequently, the slides were rinsed with phosphate buffer solution (PBS) and subsequently obstructed with 1% BSA in PBS for 1 h at ambient temperature. The sections were subjected to Immunostaining using a primary anti-caspase-1 antibody (Novus biologicals, 0.1 mL USA; Cat.No. NB100-56565) overnight at 4°C, followed by another wash with PBS. HRP-conjugated secondary antibody was then incubated with the sections, which were subsequently reacted with DAB (3, 3’-diaminobenzidine). In order to calculate the levels of positive immune-expression for caspase-1 immunohistochemical staining, six distinct fields (magnification, × 400) were chosen at random for each tissue section of every sample. The Leica application module for tissue sections analysis, connected to a Full HD microscope imaging system (Leica Microsystems GmbH, Germany), was utilized to collect morphological measurements and analyze the data. Image quantitation was carried out with image analysis software (ImageJ, 1.48a, NIH, USA) to determine the area percent (A %) [25].

### Statistical Analysis

The data was presented using the mean ± standard deviation (SD). To compare parametric data from more than two groups, analysis of variance (ANOVA) with post hoc test (Tukey’s Multiple Comparison Test) was employed. Statistical significance was determined at *P* < 0.05. The data analysis was performed using IBM SPSS statistics (V.19.0, IBM Corp., USA, 2010).

## Results

### Mortality Rate and Morphological Changes

All animals in each of the four groups successfully completed the whole experiment without any mortality. Furthermore, both the DOX group and the SEC + DOX group exhibited reduced food intake and decreased levels of physical activity. Specifically, the investigation started with a 48-h period characterized by elevated body temperature.

### The Impact of SEC on Final Body Weight, Heart Weight and Cardiac Index

In comparison to the control group, rats treated with DOX experienced less final body weight by 7.48%. Additionally, there was a significant increase in their cardiac weight and cardiac index by 25% and 35.1% respectively (*p* < 0.001). Conversely, for SEC-treated rats, their final body weight was significantly higher by 6.31% compared to the DOX group. Moreover, there was a decrease in both their cardiac weight and index by 15.1% and 20.1% respectively compared to the DOX group (p < 0.001). Finally, rats that received both SEC and DOX displayed higher final body weight by 4.62%, and their cardiac weight and index were significantly reduced by 7.1% and 11.1% respectively, in comparison to the DOX group (p < 0.001) **(**Table 2**)**.

**Table 2:** Impact of SEC on body weight heart weight and cardiac index

Tested groups	Body Weight (gm)							Heart Weight(gm)				Cardiac index × 10^3	
	Initial			Final									
Control	166.9	±	3.33	173.7	±	2.98		0.55	±	0.029		3.16	
DOX	176.0	±	3.92	160.7	±	2.81	**a**	0.69	±	0.014	**a**	4.27	**a**
SEC	166.1	±	4.45	170.9	±	4.33	**b**	0.58	±	0.032	**b**	3.41	**b**
SEC + DOX	172.0	±	6.22	168.1	±	5.96	**b**	0.64	±	0.066	**a,b,c**	3.79	**a,b,c**

Data are represented as mean ± SD (n = 7). a, b, c: Statistically significant from the control, DOX group and SEC group, respectively at P < 0.001 using one-way ANOVA followed by Tukey ‘s test for multiple comparisons

### The Impact of SEC on Abnormal ECG

**Fig. 2:**
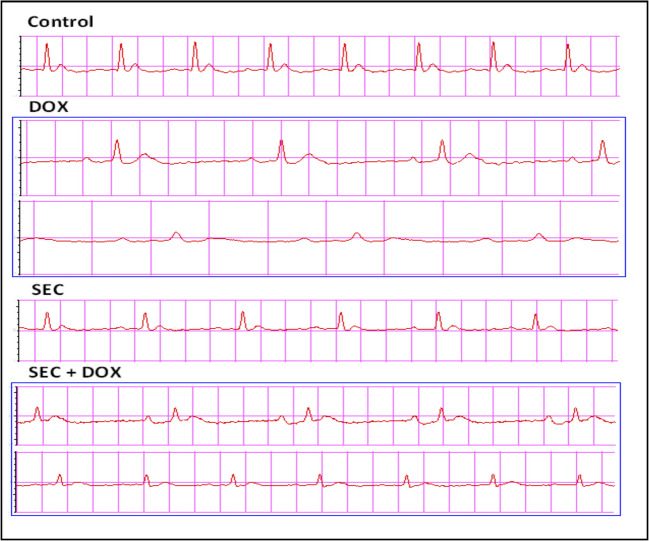
ECG graph for experimental groups in DIC: Control, DOX, SEC, and SEC + DOX groups

**Fig. 3:**
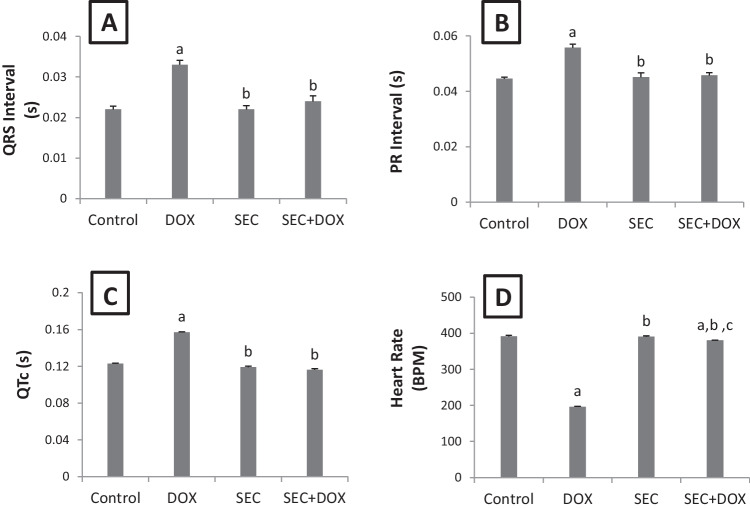
Impact of SEC on ECG abnormalities in DIC. (**A**) QRS interval, (**B**) PR interval, (**C**) QTc and (**D**) Heart rate (beat/min). Data are represented as mean ± SD (*n* = 7). a, b, c: Statistically significant from the control, DOX group and SEC group, respectively at *P* < 0.001 using one-way ANOVA followed by Tukey‘s test for multiple comparisons

Figure 2 shows ECG graph for experimental groups. Compared to the control group, the group exposed to DOX revealed a decrease in heart rate and prolonged durations of the QRS complex, PR interval, and QTc interval. However, administering SEC to intoxicated mice effectively resolved all ECG issues induced by DOX. These findings, as opposed to the DOX group, were confirmed by an increased heart rate and decreased QRS, PR, and QTc durations (Fig. 3).

### The Impact of SEC on Histopathological Alterations

**Fig. 4:**
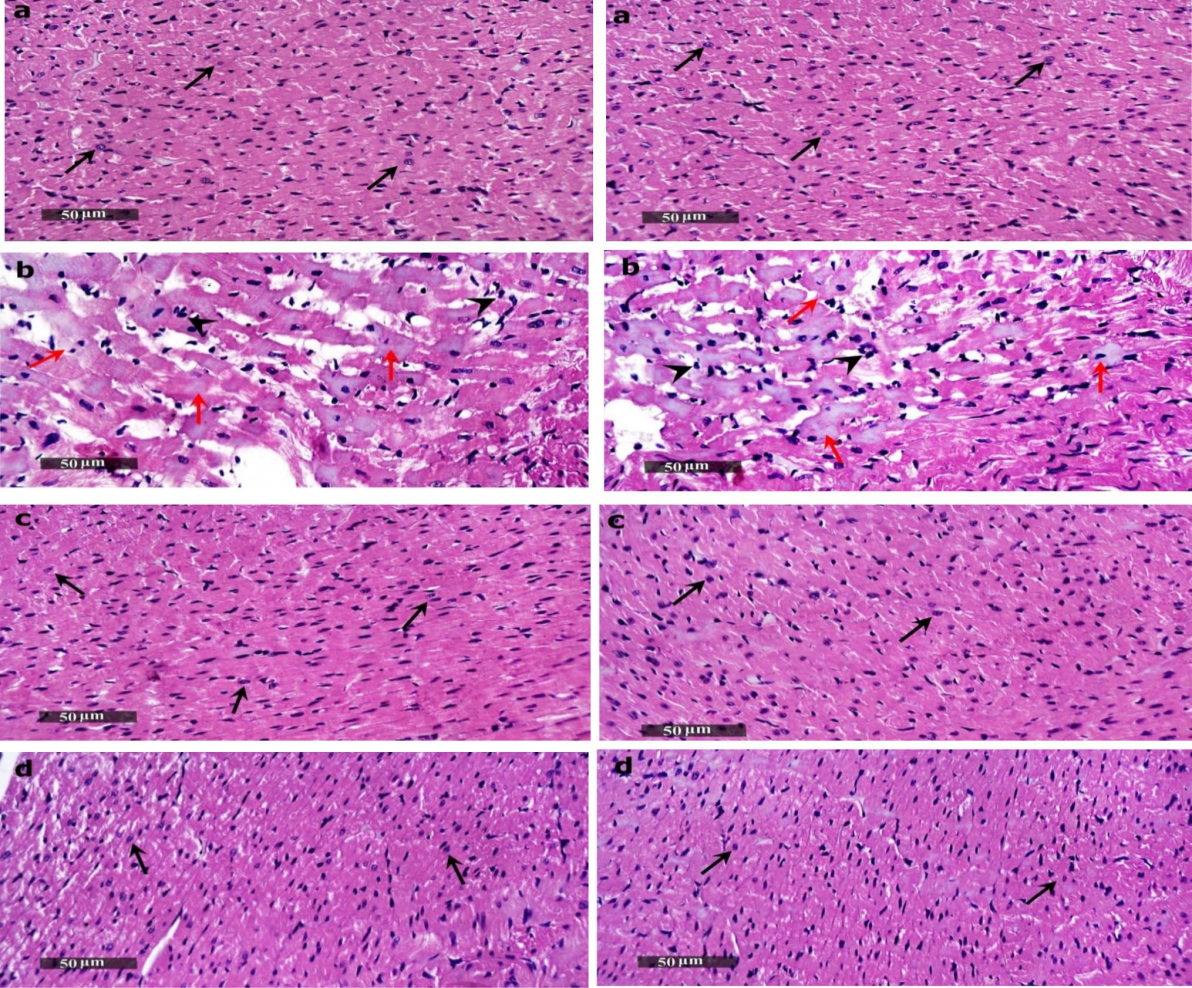
Impact of SEC on DOX-induced histological alterations of the heart tissue (*n* = 7). (**a**) Control group: demonstrated apparent intact histological structures of cardiac wall layers with almost intact branched organized cardiomyocytes with intact subcellular details (arrow) and intact vasculatures without abnormal cellular infiltrates, (**b**) DOX treated group (15 mg/kg) showed multiple focal figures of widely spaced cardiomyocytes with necrotic and degenerative changes (red arrow) accompanied with moderate inflammatory cells infiltrates (arrow head) as well as mild fibroblastic activity, (**c**) SEC (0.9 mg/kg) treated group showed the same records as control group and (**d**) SEC (0.9 mg/kg) and DOX (15 mg/kg) treated group: showed apparent intact histological features of cardiac walls with minimal abnormal degenerative changes with abundant records of apparent intact cardiomyocytes (black arrow) without abnormal infiltrates

The cardiac tissue obtained from the healthy group had well-organized cardiomyocytes with characteristic histological features. No evidence of deterioration or mortality was seen. Concerning the DOX group, observable changes in their cardiac muscle cells included deteriorating modifications, such as increased fibroblastic activity and the presence of inflammatory cells (Table 3). However, the introduction of SEC and DOX simultaneously effectively resolved these issues. The administration of SEC alone resulted in the production of well-organized cardiomyocytes that were equivalent to those seen in the control group. Based on the findings, it seems that SEC has the potential to safeguard the heart of rats from DOX-induced cardiomyopathy, as shown in **(**Fig. 4).

**Table 3:** Scores of cardiac tissue histopathological changes (scale 0–4) of studied groups

Histopathological changes	Control group			DOX group				SEC group				SEC + DOX group			
Degenerative changes	0.14	±	0.14	3.43	±	0.30	**a**	0.14	±	0.14	**b**	1.86	±	0.26	**a,b,c**
Inflammatory cells infiltrates	0.29	±	0.18	3.71	±	0.18	**a**	0.29	±	0.18	**b**	1.71	±	0.18	**a,b,c**
Fibroblastic activity	0.14	±	0.14	3.43	±	0.30	**a**	0.14	±	0.14	**b**	1.86	±	0.26	**a,b,c**

Data are represented as mean ± SEM (n = 7). 0 (negative records), 1 (mild records in less than 15% of examined tissue sections), 2 (moderate records in 15–35% of examined tissue sections), 3 (severe records in 36–50% of examined tissue sections) and 4 (very severe records in more than 50% of examined tissue sections). a, b, c: Statistically significant from the control, DOX group and SEC group, respectively at *P* < 0.001 using one-way ANOVA followed by Tukey‘s test for multiple comparisons

### The Impact of SEC on Serum Cardiotoxicity Indices

**Fig. 5:**
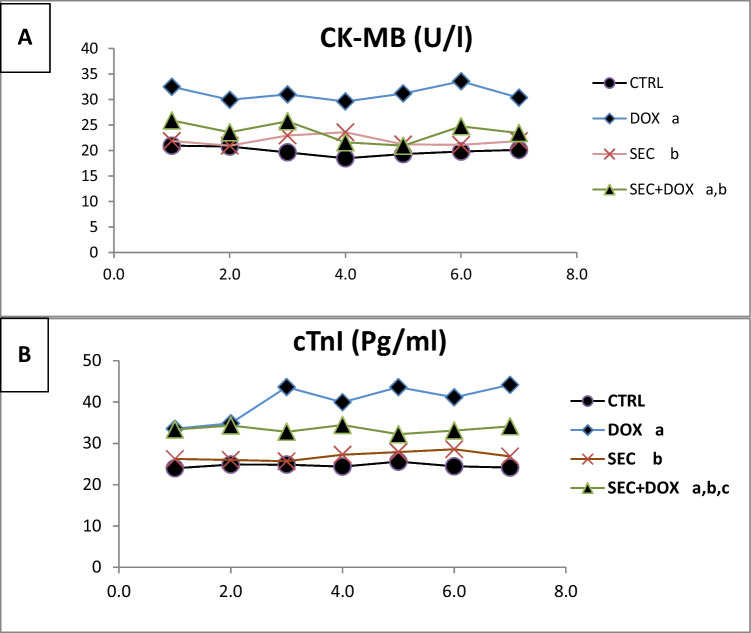
Impact of SEC on serum cardiotoxicity indices in DIC. **(A)** CK-MB and **(B)** c-TnI. Data are represented as mean ± SD (n = 7). a, b, c: Statistically significant from the control, DOX group and SEC group, respectively at *P* < 0.001 using one-way ANOVA followed by Tukey ‘s test for multiple comparisons

Blood levels of CK-MB and c-TnI were measured to ascertain the presence of myocardial injury. There was a substantial increase in serum CK-MB level by 56.9% and c-TnI level by 63.2% (*P* < 0.001) when comparing the DOX group to the control group. In contrast, the simultaneous treatment of SEC resulted in a significant decrease in serum CK-MB level by 23.9% and c-TnI level by 16.6% compared to the DOX group (*P* < 0.001), as shown in (Fig. 5).

### The Impact of SEC on DOX-Induced Oxidative Stress

The measured concentrations of MDA and GSH in the cardiac homogenate are shown in (Table 4**)**.

**Table 4:** Impact of SEC on MDA and GSH levels in cardiomyocytes

Tested groups	MDA				GSH			
	(nmol/mg total protein)				(mmol/mg total protein)			
Control	7.6	±	0.58		0.63	±	0.023	
DOX	16.5	±	2.21	**a**	0.50	±	0.007	**a**
SEC	9.2	±	1.55	**b**	0.61	±	0.064	**b**
SEC + DOX	10.5	±	1.71	**a,b**	0.58	±	0.060	**b**

Data are represented as mean ± SD (*n* = 7). a, b,: Statistically significant from the control and DOX group, respectively at P < 0.001 using one-way ANOVA followed by Tukey‘s test for multiple comparisons

The group treated with DOX showed a notable 20.4% reduction in GSH level compared to the control group, and a substantial 116.7% rise in MDA content (*P* < 0.001). In contrast, the rats which received both SEC and DOX showed a significant reduction in MDA levels (36%) and an increase in GSH levels (15.9%) compared to the group that only received DOX. This demonstrates the antioxidant capabilities of SEC, which aid in combating DIC.

### The Impact of SEC Therapy on Inflammatory Markers

**Fig. 6:**
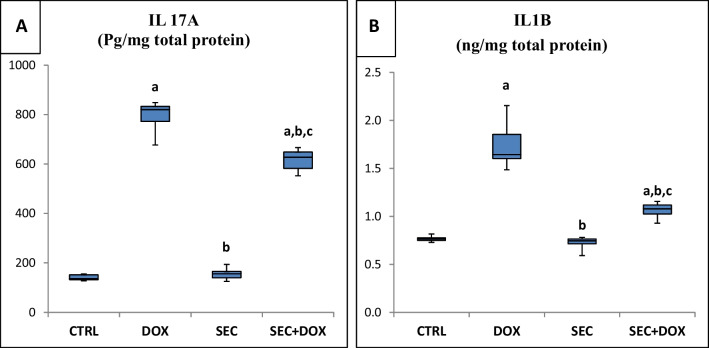
Impact of SEC on cardiac inflammatory markers in DIC. **(A)** IL-17A and **(B)** IL-1β. Data are represented as mean ± SD (*n* = 7). a, b, c: Statistically significant from the control, DOX group and SEC group, respectively at *P* < 0.001 using one-way ANOVA followed by Tukey ‘s test for multiple comparisons

The injection of DOX resulted in a considerable increase in the production of IL-17A and IL-1β by cardiomyocytes, with corresponding increases of 463% and 127.3% when compared to the control group (*P* < 0.001). The concentrations of both cytokines were markedly reduced in the SEC + DOX group in comparison to the DOX only group, with reductions of 22.5% and 38.9%, respectively (Fig. 6).

### The Impact of SEC Treatment on the Expression of the NFκB1 Gene

**Fig. 7:**
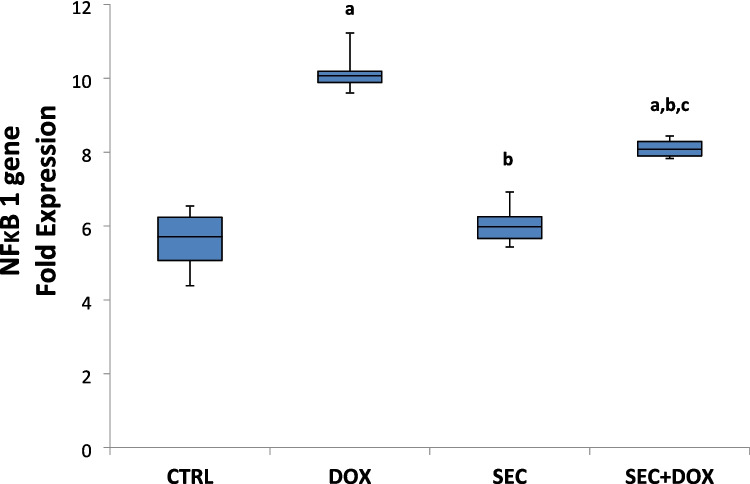
Impact of SEC on NFκB1 gene expression in heart tissue in DIC. Values are means ± SD (*n* = 7). **a**, **b**, **c**: Statistically significant from the control, DOX group and SEC group, respectively at *P* < 0.001 using one-way ANOVA followed by Tukey‘s test for multiple comparisons

Figure 7 demonstrates a significant increase in NF-κB1 mRNA expression level by 1.81-fold in rats treated with DOX compared to the control group (*P* < 0.001). When comparing the group that received only DOX with the group that received SEC co-treatment with DOX, it was observed that the latter group had a 0.79-fold decrease in NF-κB1 mRNA expression level (*P* < 0.001).

### The Impact of SEC Treatment on NLRP3 Level

**Fig. 8:**
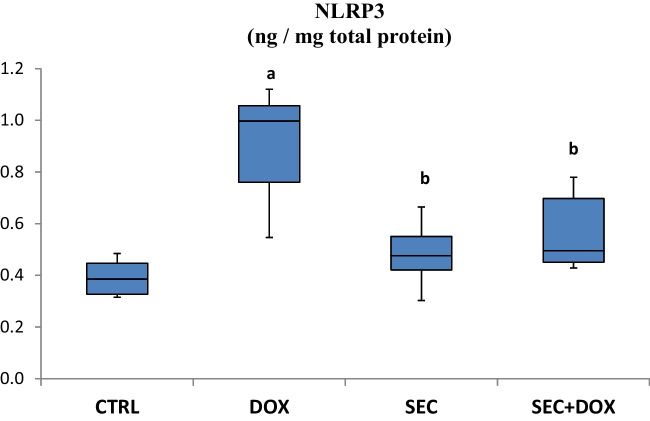
Impact of SEC on cardiac NLRP3 in DIC. Data are presented as mean ± SD (*n* = 7). **a**, **b**: Statistically significant from the control and DOX group, respectively at *P* < 0.001 using one-way ANOVA followed by Tukey ‘s test for multiple comparisons

DOX treatment resulted in a substantial 130.4% rise in NLRP3 levels in cardiomyocytes compared to the control group (P < 0.001). The addition of SEC to DOX resulted in a notable 36.5% decrease in NLRP3 levels (*P* < 0.001) as compared to the group that received just DOX (Fig. 8).

### The Impact of SEC Treatment on the Expression of Caspase-1 Protein

**Fig. 9:**
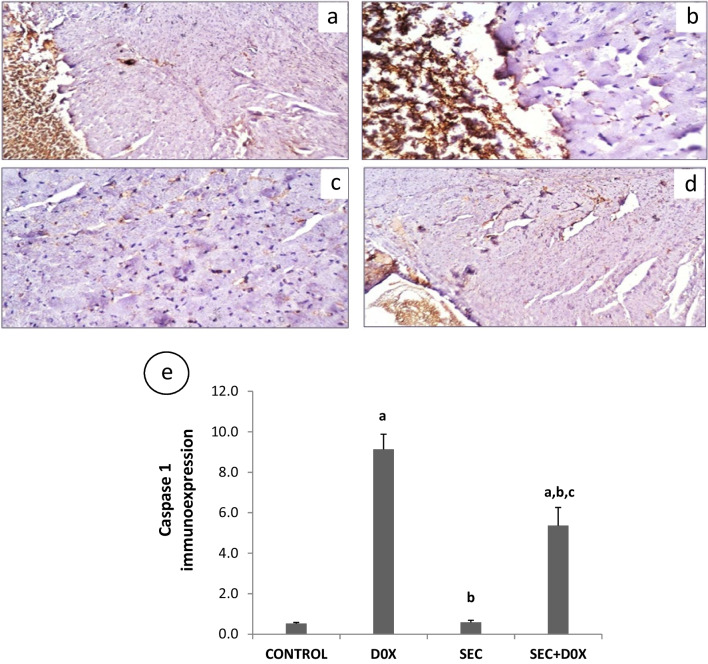
Impact of SEC on cardiac caspase-1 (40 x) in DIC. A-E: Immunohistochemical staining of cardiac caspase-1 expression. (**a**) Control group (**b**) DOX (15 mg/kg) group (**c**) SEC treated group (**d**) SEC + DOX treated group (**e**) Quantitative image analysis for caspase-1 immunohistochemical staining expressed as mean area percent. Data are presented as mean ± SD (*n* = 7). a, b, c: Statistically significant from the control, DOX group and SEC group, respectively at *P* < 0.001 using one-way ANOVA followed by Tukey‘s test for multiple comparisons

Upon DOX intoxication, the immunohistochemical analysis of caspase-1 expression demonstrated a substantial rise, indicated by the intense brown staining (164.3%), in contrast to the control group (*p* < 0.001). Conversely, pretreatment of intoxicated rats with SEC resulted in a notable reduction in caspase-1 content, as evidenced by the faint brown staining (41.2%) compared to the DOX group (p < 0.001) (Fig. 9).

## Discussion

The primary drawback of DOX, a strong anti-neoplastic medication, is its propensity to induce cardiac complications, which may vary based on the administered dose. The cardiotoxicity of this substance leads to the development of cardiomyopathy, which significantly limits its use in clinical settings [26]. Hence, it is essential to devise novel therapeutic approaches to safeguard against DIC. Exploring the use of anti-inflammatory medications in treating CVDs is an important area of study. Indeed, Wang et al. emphasized the substantial influence of inflammation on these disorders in their recent work [27]. Initiating control over the inflammatory process from the beginning is of utmost importance, and it is essential to employ an anti-inflammatory strategy in order to accomplish this objective [28]. Participation of inflammatory cytokines in the pathogenesis and development of CVDs makes them targets in therapies against CVDs. At present, mAbs therapy has provided a promising therapeutic option for CVDs [29]. Results from clinical trials have been conducted to investigate the therapeutic efficacy of mAbs in CVDs. Anti-TNF-α antibody therapy (infliximab) significantly reduces the cardiovascular risk compared with other treatments [30]. Usage of Interleukin-6 (IL-6) blockade therapies (tocilizumab and sarilumab) achieve efficacy as therapy for rheumatoid arthritis (RA) in patients at high risk of CVD [31]. The CANTOS study demonstrated that administration of Canakinumab resulted in a significant reduction of the incidence of CVD via specific neutralization of IL-1 [32].

In light of these factors, it is essential that we promptly develop specialized molecular medications that specifically target certain cytokines or their receptors. Using monoclonal antibodies to specifically target the IL-17-IL-17R pathway is promising as an effective approach for treating inflammatory autoimmune illnesses [33]. SEC is a monoclonal antibody produced by humans that inhibits the binding of IL-17A to its receptor. A research conducted by Frieder et al. has shown the long-lasting efficacy of this medicine in managing psoriasis and ankylosing spondylitis [17]. Although clinical studies have shown its efficacy in managing inflammatory illnesses [34], its influence on CVDs is still unknown. This research sought to investigate and compare the possible benefits of SEC as source of specific neutralizing antibody for IL-17A in protecting the heart from DIC.

The rats which received DOX suffered significant myocardial damage, as shown by microscopic analysis of the tissues and observation of aberrant cardiac electrical activity. The observed abnormalities were bradycardia, extended QTc, and increased PR interval duration. Furthermore, there was a notable increase in the levels of two blood cardiac biomarkers, CK-MB and cTnI. Awad et al. demonstrated that DOX administration to rats resulted in notable ECG abnormalities and a significant increase in serum cardiac markers [35]. Preceding researches [26, 34, 36] have shown that the progression of cardiac damage induced by DOX may be influenced by both oxidative stress and inflammation. Our study confirmed the findings of these prior researches by demonstrating that DOX administration to rats resulted in a substantial increase in lipid peroxidation, as seen by elevated levels of MDA in the heart, and a considerable decrease in GSH. This pronounced oxidative stress within cardiomyocytes may contribute to degenerative changes in heart tissue, such as necrosis [19].

Significantly, the concurrent administration of SEC preserved the typical structure of cardiomyocytes, cardiac conduction, and biochemical cardiac markers. Moreover, it decreased the levels of cardiac MDA and maintained the generation of cardiac GSH, which had been diminished as a result of DOX toxicity. This highlights its importance in protecting the heart from DIC. Multiple studies validate that inflammation is a pivotal factor in the progression of DIC [37, 38]. Wang et al. and Reis-Mendes et al. found that DOX may increase the levels of many inflammatory factors, such as IL-1β, IL-6, IL-8, IL-10, IL-17, and TNF-α [36, 37]. IL-17A has been described as a prominent proinflammatory factor with substantial impact. It is widely acknowledged as the most thoroughly researched member of the IL-17 family [8]. Markedly elevated cardiac levels of IL-17A were observed following DOX treatment. The interaction of IL-17A with IL-17RA has been shown to stimulate interstitial remodeling, proliferation, and inflammation through IL17R/NF-κB signaling [39], as evidenced by the upregulation of NF-κB gene expression in DOX-intoxicated rats in our study. Furthermore, our findings highlighted the robust anti-inflammatory properties of IL-17A neutralization, and its proposed ability to protect the heart from DIC, as demonstrated by the suppression of both IL-17A and NF-κB inflammatory markers.

Ucci et al. provides evidence that NF-κB is a key regulator of cellular inflammatory responses triggered by proinflammatory stimuli [40]. It has a crucial function in regulating molecular pathways linked to inflammation [41]. NF-κB signalling activation initiates the transcription of pro-IL-1β and NLRP3, which is essential for the initiation of inflammasome activity [42]. NLRP3 inflammasome activation drives inflammatory infiltration within myocardial tissues, and is a major contributor to progression of cardiovascular disorders [43]. NLRP3 is considered the most extensively studied inflammasome sensor in the heart [44], forming oligomers and binding to pro-caspase-1 via apoptosis-associated speck-like, resulting in caspase-1 activation [42]. Active caspase-1 then cleaves pro-IL-1β into its mature form, after which the activated NLRP3 inflammasome and caspase-1 are partially released from the cells along with IL-1β [45]. Doxorubicin negatively affects cardiac functions and enhances cardiac fibrosis in mice, causing elevation of cardiac expression of NLRP3, caspase-1 and IL-1β [46]. This pathway was confirmed in the current study, where administration of DOX increased the levels of NLRP3, caspase-1, and IL-1β in the heart. Conversely, co-treatment with SEC reduced their levels, indicating that the cardioprotective role of SEC in DIC may be achieved through modulation of the NF-κB/NLRP3/caspase-1/IL-1β axis.

Finally, our findings indicate that the simultaneous administration of SEC with DOX preserved serum cardiac indices and cardiac levels of GSH, while also decreasing cardiac MDA, IL-17A, and IL-1β levels. This suggests the potent antioxidative and anti-inflammatory properties of SEC. Interestingly, this study is the first investigation to highlight the role of SEC in regulating the NF-κB/NLRP3/caspase-1/IL-1β axis in DIC. Further investigations are necessary to clarify the exact mechanism of IL-17A blockage. It is advisable to conduct additional clinical trials utilizing different doses of SEC to uncover its beneficial effects in clinical environment.

## Data Availability

All supporting the findings of this report are included in this article. The data are available from the corresponding author upon reasonable request.
